# A Great Hospital for Egypt

**Published:** 1920-11-20

**Authors:** 


### A Great Hospital for Egypt.

The Egyptian Government is reported to be about to build a hospital with 1,250 beds, for the treatment of Eastern diseases, on a site of fifty acres on Rôda Is'and, Cairo. Many of these diseases are obscure, and a medical school will be attached to the institution. The design for the hospital will be chosen by competition, and the architects responsible for the twelve best designs will receive an honorarium of 500 guineas. The cost of the proposed hospital is expected to be considerably more than one million pounds.

The proposed hospital, which will be able to treat 3,000 out-patients a day as well as 1,225 in-patients, is in the hands of a committee representing the Ministries of Public Health, Public Education, Public Works, and Finance. The president is H. E. Mohammed Shafik Pasha, Minister of Public Works, and its vice-presidents are Mr. C. A. de Cossan, Director-General States Buildings Department, and Colonel Owen Richards, C.M.G., D.S.O., director of Kasr el Aini Hospital and Medical School. The latter institution, which was built about one hundred years ago by Mohammed Ali, has become insufficient, hence the decision of the Council of Ministers to establish the new hospital, and the appointment of Mr. J. W. Simpson as assessor to the Egyptian Government. After a visit to Egypt last September, he chose a site of nearly fifty acres at the northern end of Rôda Island, south of the main city and between it and Old Cairo. Here it will be accessible, free from dust, and close to the proposed new university, which the medical students will attend, and also to Kasr el Aini Hospital, part of which may be converted into laboratories. The British staff of the hospital is represented by Mr. H. B. Day and Mr. L. P. Philips, and the Egyptian staff by Dr. Azmy Bey and Neguib Bey Nahfouz.

